# Glycation and Glycosylation in Cardiovascular Remodeling: Focus on Advanced Glycation End Products and O-Linked Glycosylations as Glucose-Related Pathogenetic Factors and Disease Markers

**DOI:** 10.3390/jcm10204792

**Published:** 2021-10-19

**Authors:** Elena Dozio, Luca Massaccesi, Massimiliano Marco Corsi Romanelli

**Affiliations:** 1 Laboratory of Clinical Pathology, Department of Biomedical Sciences for Health, Università degli Studi di Milano, 20133 Milan, Italy; luca.massaccesi@unimi.it (L.M.); mmcorsi@unimi.it (M.M.C.R.); 2 Service of Laboratory Medicine1-Clinical Pathology, IRCCS Policlinico San Donato, San Donato Milanese, 20097 Milan, Italy

**Keywords:** advanced glycation end products (AGE), cardiac remodeling, diabetes, O-linked glycosylation, metabolic disorders, receptor for advanced glycation end products (RAGE)

## Abstract

Glycation and glycosylation are non-enzymatic and enzymatic reactions, respectively, of glucose, glucose metabolites, and other reducing sugars with different substrates, such as proteins, lipids, and nucleic acids. Increased availability of glucose is a recognized risk factor for the onset and progression of diabetes-mellitus-associated disorders, among which cardiovascular diseases have a great impact on patient mortality. Both advanced glycation end products, the result of non-enzymatic glycation of substrates, and O-linked-N-Acetylglucosaminylation, a glycosylation reaction that is controlled by O-N-AcetylGlucosamine (GlcNAc) transferase (OGT) and O-GlcNAcase (OGA), have been shown to play a role in cardiovascular remodeling. In this review, we aim (1) to summarize the most recent data regarding the role of glycation and O-linked-N-Acetylglucosaminylation as glucose-related pathogenetic factors and disease markers in cardiovascular remodeling, and (2) to discuss potential common mechanisms linking these pathways to the dysregulation and/or loss of function of different biomolecules involved in this field.

## 1. Introduction

**Figure 1:**
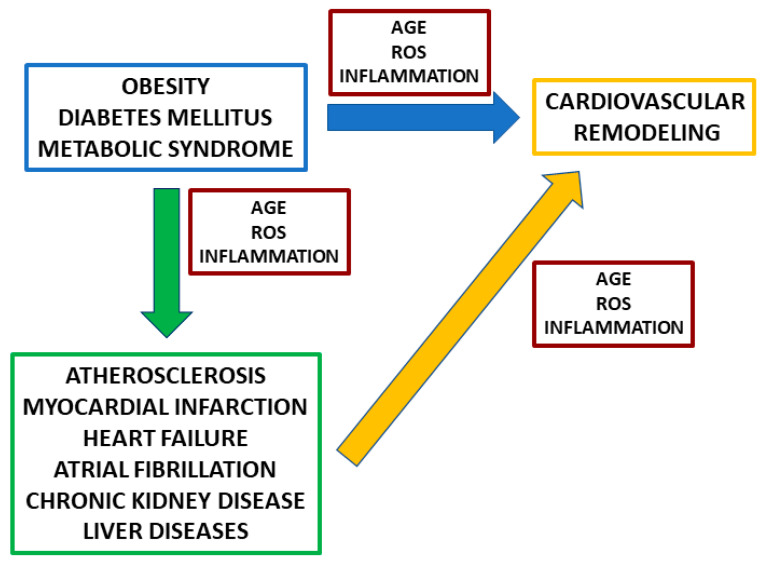
Metabolic disorders, advanced glycation end products (AGE), and cardiovascular remodeling. Obesity, diabetes mellitus, and metabolic syndrome may induce cardiovascular remodeling both directly, by inducing side effects due to the increased levels of AGE, reactive oxygen species (ROS), and inflammation, and indirectly, by promoting other disorders such as atherosclerosis, myocardial infarct, heart failure, atrial fibrillation, and kidney and liver diseases. The high levels of AGE, ROS, and inflammatory mediators that characterize these pathologies can, in turn, play additional roles in promoting cardiovascular remodeling.

Glycation and glycosylation are non-enzymatic and enzymatic reactions, respectively, of glucose, glucose metabolites, and other reducing sugars with proteins, lipids, and nucleic acids. Glycation is a random mechanism occurring at increasing free sugar availability and increased oxidative stress, two features of metabolic disorders such as diabetes mellitus (DM), metabolic syndrome, and obesity. The covalent binding of these substrates to proteins leads to the synthesis of advanced glycation end products (AGE). Glycation may impair protein function and stability and induce the synthesis of pathogenetic molecules that promote the onset and progression of different diseases, among which are cardiovascular disorders [1,2,3] (Figure 1). Furthermore, products of glycation, such glycated hemoglobin (HbA1c) and glycated albumin (GA), are used in clinical practice as biomarkers of glucose homeostasis in DM and are potential prognostic factors for DM-associated diseases [3].

Differently from glycation, glycosylation is a post-translational modification mediated by glycosyltransferases in which a defined carbohydrate molecule is added to a predetermined region of a protein. Being a controlled mechanism, its main role is to confer defined properties to living cells and it is a normal part of protein biosynthesis [4].

Although these types of protein modifications, namely glycation and glycosylation, seem to have little in common, both have been shown to promote the onset and progression of cardiovascular remodeling associated with metabolic disorders. Furthermore, redox regulation emerged as the main potential linker between these two types of reactions and associated diseases [1,5,6,7,8,9].

Cardiovascular remodeling is defined as a group of molecular, cellular, and interstitial changes that occur in the heart and vessels because of different injuries. Changes in size, geometry, and function are the key events occurring in the heart. The pathophysiology includes cell death, changes in energy metabolism, inflammation, oxidative stress, alteration in the extracellular matrix, neurohormonal activation, and changes in ions transport [10].

In this review, we aim to summarize the most recent data about the role of glycation and O-linked glycosylation, a type of glycosylation, as pathogenetic factors and disease markers in cardiovascular remodeling associated with metabolic disorders, and to discuss potential common mechanisms linking these pathways to dysregulation and/or loss of function of different biomolecules involved in this field.

## 2. Advanced Glycation End Products (AGE) and Soluble Receptor for Advanced Glycation End Products (RAGE)

AGE are the products of the non-enzymatic reaction between sugars, mainly glucose and its metabolites, with proteins, lipids, and nucleic acids. As glucose level increases, the rate of glycation increases, and AGE start to accumulate and exert damaging effects [2,11]. Although AGE synthesis is mainly due to hyperglycemia, such as in diabetes mellitus (DM), their production is accelerated in any pathological condition characterized by increased oxidative stress and inflammation, such as obesity, metabolic syndrome, as well as in many cardiovascular disorders, such as myocardial infarction and ischemia/reperfusion injury (MI/IR), vascular injuries, atrial fibrillation, and chronic kidney disease (CKD) [10]. In fact, high levels of reactive oxygen species (ROS) and inflammatory mediators can promote the synthesis of highly reactive carbonyl intermediates from lipid peroxidation, such as glyoxal and methyl-glyoxal, which lead to AGE formation through the interaction with other bioactive molecules [11,12]. Notably, AGE levels are also higher in CKD because of the reduced ability of the kidneys to remove these products and the onset of a uremic milieu [13,14]. Therefore, AGE synthesis can be considered a further marker of inflammation, which in turn, by exerting receptor-dependent and -independent effects, can promote the progression of such disorders. Although AGEs may be synthesized through different mechanisms, the damaging effects of these molecules are the same regardless of hyperglycemia. A detailed description of the mechanisms promoting AGE-related remodeling in different cardiovascular tissues can be found in the following sections. Many different proteins may be part of the substrate of AGE production and different reactions can take part in their synthesis, thus contributing to the generation of a heterogeneous group of irreversible products. AGEs are considered detrimental molecules for two main reasons: (1) They can induce modification in the structure and function of proteins involved in many processes and (2) they can activate a receptor-mediated cellular response that induces the synthesis of pro-inflammatory molecules, affects cell survival, differentiation, and proliferation, and induces metabolic changes [12].

These effects are promoted by the receptor for advanced glycation end products (RAGE). Under physiological conditions, RAGE is expressed everywhere at a low level, but in any condition characterized by increased levels of RAGE ligands, RAGE activation promotes its expression and amplifies inflammation and inflammation-related responses [13]. Besides the cell membrane form, RAGE also exists as a circulating molecule called sRAGE. sRAGE is a pool composed by cRAGE, the cleaved form of the membrane receptor, and esRAGE, the endogenously secreted form. The first one derives from the proteolytic cleavage of the membrane RAGE by metalloproteases (MMPs) [14,15], whereas the second one is an alternative splice form of RAGE [16,17]. Both forms can bind RAGE ligands, preventing their interaction with membrane RAGE and the activation of cellular responses. Nevertheless, due to the mechanisms leading to their production, their role as biomarkers is different. Being the product of MMPs, cRAGE is considered a surrogate marker of inflammation because the expression and activity of MMP enzymes are increased in inflammatory disorders. Differently, esRAGE is considered the real decoy receptor and its levels decrease after RAGE stimulation [14,18,19].

Furthermore, sRAGE and its different forms seem to play a role as diagnostic and prognostic biomarkers.

### 2.1. AGE–RAGE Pathway in Cardiac Remodeling in Different Diseases

#### 2.1.1. AGE, RAGE, and Cardiac Remodeling in Diabetes Mellitus

Continuous exposure to high glucose levels is one of the major factors inducing cardiovascular complications in DM, such as atherosclerosis, myocardial infarction, diabetic cardiomyopathy, and stroke. The AGE/RAGE pathway driven by hyperglycemia has been shown to increase the synthesis and accumulation of the extracellular matrix (ECM) at the cardiovascular level through the activation of profibrotic signaling pathways [20]. ECM accumulation may result from increased matrix protein synthesis and/or decreased degradation. This can promote alterations in fibroblasts–ECM communication and induce fibroblast differentiation into myofibroblasts [21,22]. These cells exhibit increased ECM production, contractile properties, and a reduced ability to migrate [23,24]. AGEs can induce ECM accumulation by promoting the expression and secretion of multiple types of collagens and pro-fibrotic factors, by generating ECM crosslinking, by perturbing cell–matrix interaction and cell adhesion, and by changing the expressions of proteins that regulate oxidative stress and inflammation. Some of these effects are due to the formation of AGE-modified macromolecules, as well as the activation of the AGE–RAGE axis, whose blockage was shown to improve ECM alteration [25,26].

In DM, heart failure is a consequence of left ventricular hypertrophy, which is promoted by ECM accumulation and remodeling, oxidative stress, and inflammation. AGE interaction with RAGE induces ROS formation and activates other signaling proteins, such as extracellular signal related-kinase 1/2 (ERK1/2), which in turn increases the expression of nuclear factor kappa-light-chain-enhancer of activated B cells (NF-κB) and NF-κB phosphorylation. Furthermore, AGEs can alter the expression of superoxide dismutase and impact the expression of proteins related to ECM remodeling [22,27,28,29].

It has been shown that cardiac tissue from type 2 DM patients is enriched by myofibroblasts. A recent report by Burr et al. demonstrated an elevated presence of AGEs in the diabetic ECM and the role of AGEs in promoting the fibroblast transition into myofibroblasts, as suggested by an increased expression of α-smooth muscle actin (α-SMA) [30]. Although the role of myofibroblasts in ECM remodeling is well known, as well as the intracellular signals involved in this transition, the triggers of such a transformation are still far from being fully understood. Considering that AGE/RAGE axis inhibition via pharmacological methods and the RAGE knockout model prevent myofibroblast transition and ECM stiffness, AGE accumulation appears of paramount importance to left ventricular remodeling in DM [30].

DM is also a risk factor for atrial fibrillation (AF) [31]. Since AGE and RAGE are involved in atrial structural remodeling in diabetic rats, they can promote AF. Histological and immuno-histochemical examinations of the atria of DM rats showed diffuse interstitial fibrosis, increased expression of RAGE, and connective tissue growth factor (CTGF). Pharmacological inhibition of AGE formation reduced DM-induced atrial fibrosis along with a reduction of CTGF. This observation confirmed that the AGE–RAGE system could also play a role in atrial structural changes [32].

Methylglyoxal (MO) is an intermediate of glycolysis and a precursor of AGE. Rats administered with MO showed a depletion of antioxidant enzymes, induction of fibrosis, and increased expression of RAGE and pro-inflammatory and pro-fibrotic mediators, such as transforming growth factor β (TGFβ), small mother against decapentaplegic proteins 2 and 3 (SMAD2, SMAD3), interleukin-6 (IL-6), and tumor necrosis factor α (TNFα). Retinoic acid, an active metabolite of vitamin A, prevented cardiac remodeling and fibrosis [33]. AGE infusion in rats also promoted heart fibrosis by increasing the expression of MMP-2, MMP-9, TNFα, TGFβ, RAGE, and NF-κB. The administration of gallic acid was shown to prevent AGE-induced fibrosis by regulating the expression of signaling molecules involved in this process [34].

sRAGE has been proposed as a potential biomarker of cardiac remodeling, heart failure, and its severity and mortality, and some studies indicated a strong association with N-terminal (NT)-pro hormone BNP (NT-proBNP) levels. However, most of these studies have been performed in clinical settings different form DM alone, such as hypertension, CKD and end-stage CKD, and ischemia-reperfusion injury [35,36,37,38,39,40]. In the context of DM, the increase in sRAGE levels has been regarded as a counter-regulatory system to protect against the detrimental effects of increased AGE levels and reflects the increased expression of RAGE at the tissue level [41]. One study from our group compared sRAGE levels in DM and non-DM CKD patients. sRAGE was higher in both DM and no-DM patients and was an independent predictor of BNP levels, regardless of the glycemic status. This was perhaps due to the co-existence of CKD, which affects AGE and sRAGE levels too [42]. Therefore, the role of sRAGE as an early biomarker of cardiac remodeling and a prognostic factor in DM needs further investigation.

#### 2.1.2. AGE, RAGE, and Cardiac Remodeling in Chronic Kidney Disease (CKD)

Individuals affected by DM, obesity, and metabolic syndromes have an increased risk of developing CKD [43]. In CKD, AGE and sRAGE accumulate due to their increased synthesis and reduced elimination. CKD is related to cardiovascular disease through different mechanisms, among which are the production of inflammatory mediators and ROS, the accumulation of uremic toxins, the toxicity of phosphate, and the activation of the fibroblast growth factor 23 pathway (FGF-23) [44]. The uremic milieu induced by reduced kidney function can play a pivotal role in the generation of AGE, which in turn contributes to worsening kidney function and increases cardiovascular risk and mortality [45,46]. Interestingly, patients with renal dysfunction often display diastolic dysfunction and a higher risk of developing heart failure (HF) [47,48].

AGE may induce diastolic dysfunction by activating different pathways. As previously discussed, AGE can promote tissue rigidity by directly inducing protein cross-linking. Through the activation of RAGE, AGE can stimulate intracellular pathways that lead to the upregulation of pro-fibrotic mediators, such as TGFβ, and cause a significant delay in calcium reuptake. The result is an increased duration of the repolarization phase of the cardiac contraction, which in turn can induce diastolic dysfunction [49]. In CKD, AGE can also exert detrimental effects by modulating FGF-23. Increased FGF-23 levels have been associated with left ventricular hypertrophy and the risk of HF. It has been observed that AGE may induce the expression of FGF-23 and promote cardiac remodeling [50,51].

Traditional markers of cardiovascular risk just partially explain the high risk of heart diseases in CKD, possibly because of the uremic toxins that exert damaging effects too [52]. Therefore, the need for other markers is compelling, and AGE and sRAGE might be interesting molecules to look at. Pentosidine, an AGE product, was independently linked to the mean wall thickness, relative wall thickness, and left ventricular end-diastolic volume in hemodialysis patients [53]. Elevated levels of S100A12, a RAGE ligand whose levels increase in many inflammatory disorders, were shown to promote cardiac hypertrophy and diastolic dysfunction in mice with CKD. FGF-23 produced by cardiac fibroblasts was considered a potential link between S100A12/RAGE-induced inflammation and diastolic dysfunction [50]. Although some studies suggested the role of sRAGE as a diagnostic and prognostic marker of HF [36,54,55], its role in CKD is still under debate. Previous research from our group indicated an existing positive association between sRAGE and BNP in hemodialysis and peritoneal dialysis patients, thus suggesting a role of sRAGE as a potential marker of cardiac remodeling in these patients [42]. Furthermore, in the same group of patients, the elevation of sRAGE levels was a prognostic factor for mortality. Although these data did not allow us to conclude whether sRAGE can be a marker of HF, its quantification in CKD appears to be a useful tool to identify high-risk patients. Differently, Leonardis et al. [35] showed an inverse association between sRAGE levels and clinical parameters of cardiac function, but in CKD patients not yet on dialysis. Considering that sRAGE accumulates at decreasing kidney function, its role as a biomarker of cardiac remodeling may be influenced by the clinical setting of the patients. Therefore, we need additional studies to shed light on such differences.

#### 2.1.3. AGE, RAGE, and Cardiac Remodeling after Myocardial Infarction

Myocardial infarction (MI) is one of the cardiovascular diseases occurring in patients affected by metabolic disorders [56]. The AGE–RAGE pathway can play a role in both the onset and progression of coronary disorders and MI, and in the cardiac remodeling process occurring after an acute event. Left ventricular dilatation, geometry alteration, eccentric hypertrophy, and thinning of the wall in the scar area are some of the main changes that lead to cardiac remodeling and are associated with a worse prognosis [57]. Understanding the molecular mechanisms of cardiac remodeling after MI and the identification of biomarkers of this process can improve diagnosis, risk stratification, and the development of new therapies that improve the clinical outcome of these patients.

AGE may accumulate after MI because of the inflammatory reaction and increased oxidative stress [58,59,60]. After MI, AGE can stimulate the inflammatory response in cardiac cells and promote the expression of molecules involved in the remodeling of the cellular matrix, such as MMPs [61]. During MI, both serum and myocardial levels of RAGE were higher and correlated with troponin I and creatine kinase-MB, two markers of heart damage [62]. In animal models, the intramyocardial administration of sRAGE, which works as a decoy receptor for AGE, reduced myocardial fibrosis by decreasing TGF-β1 expression, an inflammatory mediator exerting pro-fibrotic actions [63]. The blockage of cardiac RAGE expression by in vivo gene silencing in a rat model of ischemic surgery reduced inflammatory cytokine release, apoptotic cells, infarct size, and fibrotic tissue formation [64]. Considering that the size of MI influences ventricular function, RAGE silencing displayed important cardioprotective effects against post-MI cardiac remodeling. Fracasso et al. [65] characterized AGE formation and RAGE expression in both plasma and cardiac tissue after MI in rats. Differently from previous studies, no increase in plasma AGE or sRAGE was observed, and a decrease in AGE and an increase in RAGE levels were found in the myocardium. The authors concluded that the rat MI model is not a useful model to explore the clinical observations previously described.

Different molecules related to cardiac remodeling can interact with the AGE–RAGE pathway. Galectin-3 is expressed in many tissues and its cardiac levels increase early when the cardiac remodeling starts to occur, especially after MI [66,67]. Galectin-3 has been shown to modulate many different pathways that lead to HF, such as fibroblast proliferation and ECM turnover, and its plasma levels correlated with established markers of cardiac remodeling [68,69]. Interestingly, galectin-3 facilitates AGE interaction with RAGE, thus also contributing to the exacerbation of cardiac remodeling through the cooperation with this pathway [70]. The extracellular high-mobility group box-1 protein (HMGB1), a danger signal released from necrotic cells, is involved in tissue repair and host defense. HMGB1 can modulate pro-inflammatory and pro-fibrotic responses via RAGE in ischemia/reperfusion (I/R) injury [71,72]. The vascular endothelial growth factor (VEGF) is crucial for myocardial angiogenesis and myocardial salvage after MI, and its plasma levels increase in patients with MI and in an experimental rat model of MI [73,74,75]. Interestingly, S100B, an intracellular calcium-binding protein that is released after MI from damaged myocytes, can activate post-MI remodeling by inducing VEGF upregulation through RAGE activation [76,77].

The role of plasma AGE and sRAGE as early biomarkers of cardiac remodeling post MI are under debate. In the work by Raposeiras-Roubin et al., plasma AGEs were an independent and predictive biomarker of HF at 1 year after MI, regardless of age, presence of DM, infarct severity, and other markers [78]. Differently, Redondo at al. observed no correlation between AGE and the left ventricular ejection fraction at 6 months after MI, and sRAGE was not a predictor of left ventricular end-diastolic or -systolic volumes [79]. However, the AGE/sRAGE ratio was directly related to left atrium increase, thus suggesting that the levels of sRAGE can prevent the detrimental effect of AGE on atrial remodeling. sRAGE levels resulted as a better marker of atrial increase compared to ventricular remodeling [79].

#### 2.1.4. AGE, RAGE, and Atrial Fibrillation (AF)

AF is a disturbance in the cardiac rhythm. Recent evidence indicated that inflammation, oxidative stress, and increased AGE levels, which are typical of metabolic disorders, can promote AF [80,81]. Patients affected by DM and/or obesity display an increased risk of developing atrial fibrillation too [56]. Considering the roles of AGE in inducing protein crossing and that of AGE–RAGE axis in promoting inflammation, oxidative stress, and finally fibrosis, these findings seem to suggest a potential association between AGE and AF [82]. In addition to structural remodeling of the atrium, AGE can also promote electrical remodeling. It was supposed that fibrosis induces early afterdepolarization and triggers activity at the level of the left atrial pulmonary vein junction due to the increased diastolic calcium [83]. ROS, which are produced after RAGE activation, prolong action potential duration, and stimulate L-type calcium current, which in turn facilitates early afterdepolarization [84]. This last factor can in turn increase late sodium current, thus leading to AF [85]. The overexpression of inflammatory mediators, such as TNF-α, prolongs the action potential and calcium transient duration [86].

sRAGE might be a useful marker in AF; however, conflicting results exist regarding its role. The different results may deal with the type of sRAGE form that has been quantified and the clinical features of AF patients. Raposeiras-Roubin at al. [87] observed a clear relationship between AGE, total sRAGE levels, and AF, and a positive correlation with left atrial volume and area. These results were reported to be independent of DM, which is one of the leading causes of increased AGE levels. AGE inhibition was also proposed as a therapeutic target for atrial remodeling and AF. Zhao et al. [88] evaluated the role of esRAGE and cRAGE as biomarkers of atrial fibrillation in paroxysmal (if AF terminates spontaneously) and persistent AF patients. Left atrial diameter, HMGB1, the high-sensitivity C reactive protein (hsCRP), and cRAGE were higher in persistent AF than paroxysmal and control groups, while esRAGE was lower. Except for left atrial diameter, all the other parameters were also higher in paroxysmal patients than controls. These results suggest a pro-inflammatory response that activates RAGE, RAGE cleavage into cRAGE, and esRAGE down regulation. Similarly, Yan et al. [89] observed an association between the occurrence of AF, increased hsCRP, and decreased esRAGE levels. In the study by Lancefield et al., sRAGE and esRAGE independently predicted persistent compared to paroxysmal AF [90]. Al Rifai et al. reported no association between sRAGE and AF risk [91], whereas Yang et al. showed that in DM patients, sRAGE levels were associated with low recurrence of AF after catheter ablation [92]. Patients with AF have elevated AGE levels not only in plasma but also in atrial tissue [93]. In streptozotocin-induced DM rats, the inhibition of AGE synthesis reduced RAGE activation, the generation of pro-inflammatory mediators, and fibrosis, and prevented AF [32]. These different results reinforce the importance of evaluating not only total sRAGE, but also the different forms. In fact, considering that inflammation promotes membrane RAGE expression and its cleavage, the association between increased sRAGE levels and AF could mainly be due to the total cRAGE level increase.

#### 2.1.5. AGE, RAGE, and Vascular Remodeling

Increased levels of AGE are also an important risk factor contributing to vascular pathology [94]. The mediators induced by RAGE activation, such as TNF-α, can promote harmful effects by affecting the function of different cells of the vascular wall, i.e., the increased recruitment of monocytes into endothelial cells (ECs) and proliferation of vascular smooth muscle cells (VSMCs) are key events concurring in atherosclerosis and restenosis after percutaneous coronary intervention [95,96]. Although we know that inflammation is the trigger of vascular disorders, the underlying mechanisms are still far to be fully understood. EC permeability is a cellular process crucial for vascular pathology. Membrane injury is common in cells that work under mechanical stress, such as ECs of the vascular system and cardiac muscle myocytes. Membrane resealing involves different proteins that are calcium sensors, including annexins, synaptoglobins, and other cell-surface glycoproteins. Improper activity of these proteins may lead to impaired membrane resealing. Upregulation of RAGE has been suggested as one potential mechanism impairing cell membrane resealing by promoting the aggregation of cell surface glycoprotein and by forming homodimers or oligomers itself that reduce membrane plasticity. Xiong et al. demonstrated a negative role of RAGE in membrane repair. By using human umbilical vein EC expressing RAGE, they observed that RAGE expression increases the levels of β-catenin, which in turn decreases F-actin stress fibers and prevents the repair of the EC membrane in response to stress [97]. Flow-mediated vasodilatation is mainly due to the release of relaxing agents, such as nitric oxide (NO). A remodeling process, which is characterized by diameter enlargement, medial hypertrophy, and improvement of contractility, can be observed in response to a chronic flow change. In DM, the reduced ability of vessels to adapt to chronic changes in blood flow can in part explain the vascular damage observed in DM and obesity. In fact, the increased production of AGE and ROS affects the ability of arteries to adapt to chronic stress, preventing remodeling and dilation [98]. Hyperlipemia and oxidative stress can also induce AGE accumulation and RAGE up-regulation in macrophages, smooth muscle cells, and EC in the aortic and coronary lesions of hyperlipidemic rabbits prone to MI [99]. Coronary arteries in DM patients are typically described as smaller and undergo negative remodeling. Increased circulating GA, a product of glycation of albumin, and decreased esRAGE levels in serum negatively correlated with coronary artery remodeling in type 2 DM patients. Therefore, both these molecules could serve as novel biomarkers of vascular remodeling in DM [100]. AGE may also induce arterial stiffness through the activation of pathways that lead to osteogenic differentiation and calcification of VSMC. Arterial stiffness is an independent predictor of left ventricular hypertrophy and other cardiovascular diseases, and it is exacerbated by metabolic syndrome, obesity, and DM [101]. AGE crosslink breakers emerged as potential candidates to reduce arterial stiffness [102]. Both in vitro and animal studies indicated that AGE–RAGE signaling promotes vascular calcification [103,104]. By binding to RAGE, AGE can promote vascular calcification via an oxidative stress pathway [105]. In addition to promoting oxidative stress, the accumulation of AGE in the vascular wall increases RAGE expression and the activation of inflammation and pro-osteogenic signaling pathways. Since the blockage of RAGE and ROS reduced VSMC calcification, inhibition of the AGE–RAGE–oxidative stress pathway has been suggested as an effective therapy for the prevention of vascular calcification in DM [105]. Furthermore, since AGEs form irreversible cross-links of collagen and elastin, AGE-modified collagen is less sensitive to enzymatic degradation and AGE-modified elastin is more prone to calcium binding. All these modifications result in ECM stiffness and media calcification [105,106,107,108].

By using a co-culture system with THP-1 monocytes, aortic VSMC, and umbilical EC, Mudau et al. investigated the effect of the AGE–RAGE axis on EC dysfunction, as an early sign of atherogenesis, VSMC proliferation, and dysfunction [95]. AGEs were shown to directly activate a pro-inflammatory response in EC and to indirectly affect VSMC proliferation and vascular remodeling by the activation of RAGE at the endothelial level. AGEs are therefore involved in the crosstalk between EC and VSMC dysfunctions. The treatment of adult rat aortic VSMC with AGE or other RAGE ligands, such as S100B, at concentrations detected in DM patients increased markers of inflammation and apoptosis. These effects were attenuated by the concomitant administration of sRAGE [109]. Considering the damaging role of AGE and other RAGE ligands, these molecules, along with sRAGE, have been suggested as potential diagnostic and/or prognostic markers of DM-induced vascular complications. Vascular disorders may also be due to the proliferation of VSMC. RAGE expression in VSMC is low in normal vessels but enhanced after injury and exposure to RAGE ligands [110,111]. Furthermore, RAGE activation has been linked to other features of vascular remodeling, such as VSMC proliferation and resistance to apoptosis through the activation of the oncoprotein Pim1 [112,113]. Another mechanism that can contribute to vascular complication in DM is the AGE-induced modulation of MMPs and the tissue inhibitor of MMPs (TIMP). MMPs are enzymes involved in ECM remodeling, and the plasma levels of different MMPs were associated with markers of arterial stiffness and macrovascular complication in DM [114,115].

## 3. O-Linked Glycosylation

**Figure 2:**
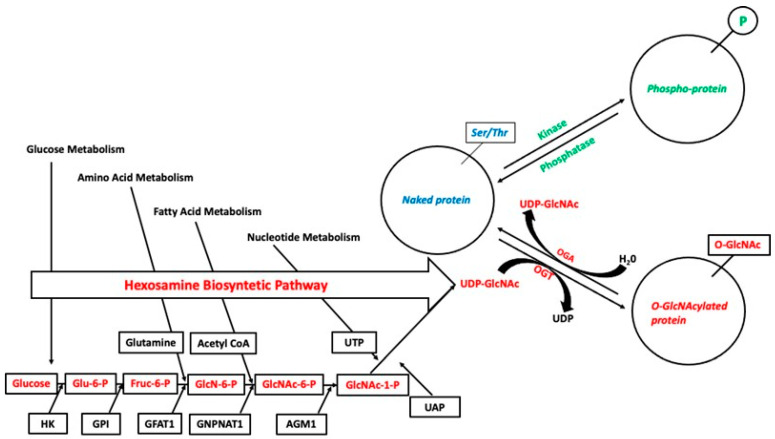
The Hexosamine Biosynthetic Pathway (HBP) and dynamic interplay between O-GlcNAc and O-phosphate. Once in the cells, glucose is enzymatically metabolized by Hexokinase (HK) to Glucose-6-Phosphate (Glc-6-P), which is then converted by Glc-6-P isomerase (GPI) into Glc-6-P to Fructose-6-phosphate (Fruc-6-P). About 3–5% of Fruc-6-P is metabolized to Glucosamine-6-phosphate (GlcN-6-P) by Glutamine Fructose-6-phosphate AmidoTransferase (GFAT). Glucosamine-6-Phosphate N-Acetyltransferase 1 (GNPNAT1) utilizes acetyl-CoA to convert GlcN-6-P into N-Acetylglucosamine-6-Phosphate (GlcNAc-6-P), which is then converted by Phospho-Acetyl Glucosamine Mutase 1 (AGM1) into N-acetylglucosamine-1-phosphate (GlcNAc-1-P). By utilizing Uridine Triphosphate (UTP), UDP-N-Acetylglucosamine Pyrophosphorylase (UAP) converts GlcNAc-1-P to Uridine diphosphate N-acetylglucosamine (UDP-GlcNAc). O-GlcNac modification and phosphorylation are reciprocal events because the site of attachment is the same or is adjacent to Ser/Thr residues. Two highly conserved enzymes mediate the addition and removal of the UDP-GlcNAc: OGlcNAc transferase (OGT) and O-GlcNAcase (OGA), respectively.

Once entering the cell, glucose is phosphorylated to glucose-6-phosphate. Then it is metabolized to fructose-6-phosphate to gain access to glycolysis and accessory pathways of glucose metabolism, including O-linked-N-Acetylglucosaminylation (O-GlcNAcylation). The hexosamine biosynthesis pathway (HBP) metabolizes a small portion of glucose to uridine diphosphate N-AcetylGlucosamine (UDP-GlcNAc), the substrate for protein O-GlcNAcylation, and other forms of protein glycosylation (Figure 2). The UDP-GlcNAc production is a process that, in addition to glucose, uses other metabolic substrates such as amino acids, fatty acids, and nucleotides. Therefore, the fluctuation in the availability of UDP-GlcNAc, which depends on the trends of different metabolic pathways, makes the variations of O-GlcNAc levels a real nutritional and metabolic sensor. Furthermore, UDP-GlcNAc levels may increase not just due to nutritional reasons, but also in any situation of increased glucose flow associated with cellular stress [116].

O-GlcNAcylation is a post-translational protein modification because of the attachment of a single N-AcetylGlucosamine (GlcNAc) molecule to residues of serine or threonine on nuclear, cytosolic, and mitochondrial proteins [117]. O-GlcNAcylation is, in many aspects, analogous to phosphorylation: (1) they are both highly dynamic and respond to various physiological stimuli, and (2) they are very close to each other since they can occur on the same or adjacent residues with antagonistic effects in the regulation of many cellular processes, such as transcription, cell signaling, and metabolism [118]. Unlike phosphorylation, which is regulated by multiple kinases and phosphatases, just two specific enzymes tightly control O-GlcNAcylation: O-GlcNAc transferase (OGT), which catalyzes the addition of GlcNAc to the hydroxyl group of serine or threonine residues, and O-GlcNAcase (OGA), which removes GlcNAc from proteins [119].

OGT and OGA activities strongly depend on the availability of UDP-GlcNAc levels, which means that they can receive information from a wide range of nutritional signals and stress pathways that modulate O-GlcNac cellular levels. It has been suggested that OGT may use its N-terminal tetratricopeptide repeat domain as scaffolding to improve interactions with its substrates [120]. The same domain undergoes several post-translational modifications that regulate its activity, including phosphorylation by signaling molecules such as the 5’ AMP-activated protein kinase (AMPK), Ca^2+^/calmodulin-dependent protein kinase II (CamKII), Checkpoint kinase 1 (Chk1), and the insulin receptor [121]. OGT and OGA are also regulated by a series of extremely complex mechanisms at both transcriptional and post-transcriptional levels. It has been shown that OGT and OGA levels compensate each other: OGT knockout or inhibition is associated with a reduction in the levels of OGA [122]. The OGA gene is located within the highly conserved NK homeobox gene cluster. Since this region is a target of the Polycomb-group complex (PcG), which is also composed by OGT, it is plausible that OGT modulates OGA expression at the transcriptional level [123]. On the other side, OGA has been shown to act as a co-activator that directly promotes OGT transcription through the cooperation with C/Ebpb (CCAAT-enhancer-binding proteins) and p300 histone acetyltransferase [124]. The OGT gene contains a conserved intronic splicing silencer that is necessary for intron retention. This retention is regulated in response to O-GlcNAc levels. If the levels are high, intronic retention takes place to avoid the further expression of the enzyme. In contrast, when O-GlcNAc levels decrease, the intron is directed to splicing and OGT can be produced [125]. The basic aspects of OGA regulation are, instead, less known. OGA can be O-GlcNAcylated at serine 405, thus acting as a substrate for OGT, but the implications of O-GlcNAcylation on OGA are still unexplored [126]. It is well known that O-GlcNAcylation is a key mechanism involved in the regulation of numerous cellular processes, both in physiological and pathological conditions. To ensure cellular homeostasis, the level of O-GlcNAcylation is maintained in a specific range, thanks to the mutual regulation of OGT and OGA at both transcriptional and post-translational levels [127]. A disruption in O-GlcNAcylation homeostasis has been associated with the pathogenesis of several diseases, including metabolic-related heart disorders such as cardiomyopathy, heart failure, hypertrophy, endothelial dysfunction, oxidative stress, ischemia, and hypoxia [128,129,130,131,132].

### 3.1. O-Linked Glycosylation in Cardiovascular Remodeling in Different Diseases

#### 3.1.1. Effect of Hyperglycemia on O-GlcNAcylation in the Heart

In the heart, O-GlcNAcylation is recognized as an important mechanism involved in the regulation of many cellular processes, including cell metabolism, mitochondrial function, protein quality control and turnover, autophagy, and calcium handling. One of the first studies reporting the effects of O-GlcNAcylation on heart proteins was published in 1996 and suggested a potential protective role of O-GlcNAcylation, regardless of hyperglycemia. In this paper, Roquemore et al. showed that, in the rat heart, the small heat shock protein alpha B-crystallin, also called HspB5, is a target of O-GlcNAcylation. In the heart, alpha B-crystallin reduces the aggregation of actin filaments (i.e., paracrystals), thus playing a protective role during acute stress conditions such as ischemia. O-GlcNAcylation allows alpha B-crystallin recycling more rapidly than the peptide itself and represents additional regulatory control in the heat-shock response [133].

Studies conducted in rats showed that OGT activity was greater in the heart than other tissues and indicated that O-GlcNAcylation of intracellular proteins may be a mechanism mediating glucose toxicity [134]. Under hyperglycemic conditions, increased mitochondrial superoxide production promotes HBP activity and O-GlcNAcylation of different substrates, such as transcription factor Sp1, which can play a role in the onset and progression of DM complications [135]. Sp1 is necessary for the correct regulation and expression of glutathione S-transferase P1 (GSTP), an antioxidant system involved in the defense against xenobiotics, oxidative insults, and cancer-causing agents [136]. Considering that O-GlcNAcylation of the transcription factor Sp1 reduces GSTP expression in the liver, we can suppose that the same mechanism might also result in a reduced response to oxidative insults in the heart [137].

The chronic increase in O-GlcNAcylation occurring in DM has been associated with cardiomyopathy [138,139,140]. Prakoso and co-workers showed a significant increase in the levels of O-GlcNAcylation, OGT, and OGA proteins in left ventricular biopsies from DM patients undergoing coronary bypass surgery in comparison to non-DM. Furthermore, the total O-GlcNAcylation level correlated directly with glycemia and HbA1c and inversely with the left ventricular ejection fraction [141]. These findings seem to suggest that the increased levels of UDP-GlcNac and O-GlcNac, resulting from hyperglycemia, may be associated with impaired heart function. A study performed in diabetic mice showed, instead, that adenovirus-mediated overexpression of OGA improves contractile properties of the heart [142]. Perhaps, O-GlcNAcylation of cardiac proteins can exert protective or harmful effects depending on the timing of activation, namely short-term or chronic increase, respectively. This needs further investigation.

#### 3.1.2. O-GlcNAcylation and Ischemia-Reperfusion (I/R) Injury

As seen above, chronic, and sustained O-GlcNAcylation of cardiac proteins is associated with several alterations of cellular metabolism, which in turn lead to deleterious effects on cardiovascular function. On the other side, transient elevation of O-GlcNAcylation of heart proteins can exert cardio-protective effects, as observed in myocardial I/R injury.

In the heart, transient O-GlcNAcylation of proteins seems to have a protective role against possible reperfusion damages. In rat ventricular myocytes, increased O-GlcNAcylation levels, due to the overexpression of OGT or increased HBP flow, improved cell viability and mitigated necrosis and apoptosis resulting from I/R lesions. A significant correlation between protein O-GlcNAcylation and cell survival was also highlighted [143,144]. Myocardial ischemic preconditioning is a protective mechanism in which the myocardium is made resistant to the deleterious effects of prolonged ischemia by exposure to brief periods of sublethal ischemia [145]. Both ex vivo and in vivo studies indicated that ischemic preconditioning increases O-GlcNAcylation of cardiac proteins with a consequent myocardial infarct size reduction [146,147]. Furthermore, plasma dialysate taken from healthy volunteers subjected to ischemic preconditioning had beneficial effects on human isolated atrial trabeculae subjected to I/R injury [148]. Since HBP pathway inhibition eliminated these beneficial effects, hemodynamic recovery was assumed to be associated with the acute elevation of O-GlcNAcylation of cardiac proteins. Acute/short-term O-GlcNAcylation has protective effects against I/R injury, also by regulating mitochondrial proteins. The voltage-dependent anion channel (VDAC) is the most abundant mitochondrial outer membrane protein and a component of the Mitochondria Permeability Transition Pore (mPTP) protein complex. In addition to regulating the traffic of mitochondrial metabolites, VDAC is involved in mitochondria-induced apoptosis through the release of the pro-apoptotic factor cytochrome C into the cytosol [149]. O-Glycosylation of VDAC exerted cardioprotective effects due to the inhibition of mPTP opening and the consequent release of apoptotic factors [150,151].

Ngoh and colleagues [152] demonstrated that, in ischemia, the cardioprotective effects of high O-GlcNac levels were also due to the reduction of endoplasmic reticulum (ER) stress, as evidenced by a decrease in the activation of the homologous protein CCAAT/enhancer-binding protein, an ER stress marker. Zafir et al. [153] observed that murine heart stem cells suffer post-hypoxic damage after OGT inhibition or OGT genic silencing, which induces a decrease in protein O-GlcNacylation. On the other side, they observed increased vitality of cardiac stem cells at increasing O-Glycosylation levels.

This protective condition, resulting from an acute and transient increase in cardiac O-GlcNAcylation, is lost in conditions of chronic/sustained O-GlcNAcylation. DM exacerbates myocardial I/R injury, due to the hyperglycemia- and hyperinsulinemia-induced up-regulation of O-GlcNAcylation [154]. Mitochondrial acetaldehyde dehydrogenase 2 (ALDH2) is an important cardioprotective enzyme whose activity inversely correlates with infarct size by promoting the detoxification of cytotoxic aldehydes. High glucose levels and therefore excessive O-GlcNacylation of ALDH2 promotes toxic aldehyde accumulation and cell apoptosis, thus representing a mechanism for the hyperglycemic exacerbation of myocardial I/R injury. The removal of O-GlcNAc from ALDH2 by an ALDH2 activator improved cardiac function and decreased infarct size and cardiac apoptosis [155]. Increased O-GlcNacylation of mitochondrial proteins is an additional mechanism linking hyperglycemia, oxidative stress, impaired mitochondrial function, and increased vulnerability to stress-induced cell death [156].

Although these data seem to be conflicting, the different results can be easily explained by simply considering the existence of the range and timing of O-GlcNAcylation at which cellular homeostasis is maintained [127]. Any change occurring in the levels of protein O-GlcNAcylation, both in excess or in defect, and the duration of O-GlcNAcylation, acute or chronic, can lead to alterations in cellular functions and can be a key factor in the pathogenesis of several diseases.

#### 3.1.3. O-GlcNAcylation and Cardiac Remodeling

It is well known that hyperglycemia is the main cause of cardiovascular complications in DM, and, as previously discussed, the activation of different metabolic pathways, such as the polyol pathway, leads to the production of reactive intermediates that contribute to the generation of AGE, oxidative stress, and inflammation [105,157]. In this scenario, superoxide overproduction is a trigger of HBP, which in turn increases protein O-GlcNAcylation [135,140]. Therefore, the synergistic action of all the factors and events listed above leads to an increase in the oxidative stress in the cardiovascular system and promotes DM-related cardiovascular complications [158,159].

During the last decade, it has been recognized that O-GlcNAcylation can play a role in cardiac myofilaments regulation. Several O-GlcNAcylation sites have been identified in cardiac myofilaments, such as α-actin, myosin heavy chain, myosin light chain, cardiac troponin I, and the myosin-binding protein. Furthermore, increased GlcNAc levels in myofilaments from cardiac trabeculae decrease cardiac myofilament sensitivity to calcium [160]. Gelinas and colleagues studied the involvement of O-GlcNAcylation of cardiac troponin T (cTnT) in ischemic heart failure and cardiac remodeling. They observed that the activation of AMPK leads to the reduction of O-GlcNAcylation of cTnT and prevents an angiotensin II-induced increase in left ventricular hypertrophy and cardiomyocyte size in wild-type mice [161]. An intriguing study performed in animal models with MI indicated the worsening of cardiac function at increasing O-GlcNAcylation of cTnT at Ser 190 and a decrease its phosphorylation at Ser 208 [162]. This suggests that O-GlcNAcylation and phosphorylation can interact with each other superbly and play a role in the onset of cardiac diseases.

#### 3.1.4. O-GlcNAcylation and Atrial Fibrillation (AF)

AF is the most common clinical arrhythmia. It is associated with significant mortality and morbidity, and DM represents a consistent risk factor for its onset and progression [163,164,165]. It is well known that ROS and O-GlcNAcylation levels are dramatically increased in atria from DM patients [148] and they can be involved in DM-associated cardiomyopathy and AF [139,166].

**Figure 3:**
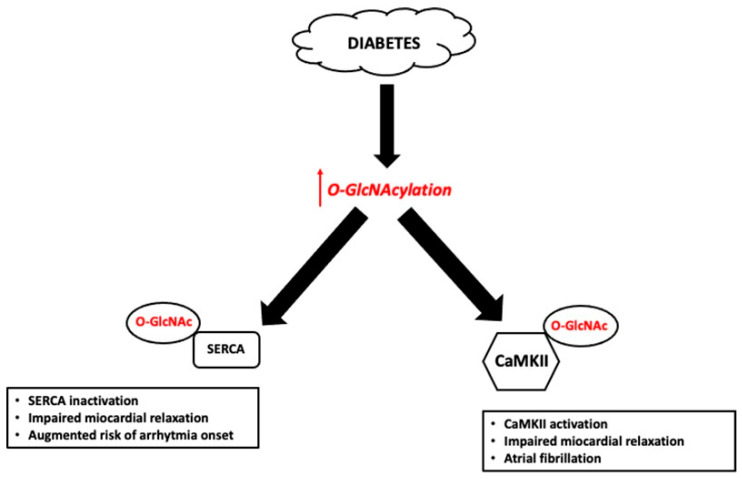
Pathological effects of O-GlcNAcylation on Sarco/endoplasmic reticulum Ca^2+^-Atpase (SERCA) and Ca^2+^/calmodulin-dependent protein kinase II (CaMKII). High levels of protein O-GlcNAcylation strongly reduce SERCA activity leading to impaired myocardial relaxation and/or augmented risk of arrhythmia onset. Conversely, O-GlcNAcylation of CaMKII (at Ser 280) leads to CaMKII activation with consequent alteration in myocardial relaxation and onset of atrial fibrillation.

Yu et al. [167] observed that, in streptozotocin-induced diabetic rats, the onset of ventricular arrhythmias is extremely frequent and potentially associated with the increased O-GlcNAcylation of cardiac voltage-gated sodium channels. The observation that the onset of these arrhythmias is countered by inhibiting Glutamine Fructose-6-phosphate AmidoTransferase (GFAT), a key enzyme in HBP, confirms the association between O-GlcNAcylation and arrhythmias [139]. The negative effect of a chronic increase in O-GlcNAcylation has also been seen on Sarco/endoplasmic reticulum Ca^2+^-Atpase (SERCA), the main carrier of calcium from the cytosol to the sarcoplasmic reticulum that facilitates cardiac relaxation. Increased O-GlcNAcylation levels reduce the expression and the activity of SERCA in neonatal rat cardiomyocytes and alter the excitation–contraction coupling [129]. The full functionality of SERCA is modulated by the phospholamban protein that is active if phosphorylated. It has been demonstrated that O-GlcNAcylation of phospholamban reduces SERCA activity, thus leading to impaired myocardial relaxation with a consequent increased risk of arrhythmias [138,168] (Figure 3). Ca^2+^/calmodulin-dependent protein kinase II (CaMKII) mediates physiological responses arising from acute β-adrenergic activation [169] and is considered a key signaling molecule in myocardial hypertrophy, heart failure, and AT [170,171]. In DM, CaMKII can be activated by O-GlcNacylation at Ser 280 and ROS-mediated oxidation, two events that have been shown to induce arrhythmia and increase post-infarction mortality [172,173,174]. The hypothesis of synergistic action of ROS and O-GlcNacylation on the CaMKII regulatory domain is supported by the fact that the target of these reactions forms a cluster of contiguous amino acids (Ser 280, Meth 281 and 282). Therefore, modulation of CaMKII may be one mechanism linking O-GlcNacylation and AT in DM [175] (Figure 3).

#### 3.1.5. O-GlcNAcylation and Vascular Remodeling

An increase in O-GlcNActylation produces contrasting effects on vascular tissue and function depending on the timing of response activation. Many studies demonstrated that an acute and transient increase in the O-GlcNAcylation of vascular proteins exerts important protective effects, as previously described in I/R injury. Inflammation and immune cell infiltration in the injured vascular walls contribute to vascular remodeling. Treatments with glucosamine (GlcN), an amino sugar that increases the production of UDP-GlcNAc and O-GlcNAcylation of proteins, and O-(2-acetamido-2-deoxy-d-glucopyranosylidene) amino-N-phenylcarbamate (PUGNAc), a competitive inhibitor of OGA, induce an acute and short-term increase in O-GlcNAc modification of protein and inhibit inflammation and neointimal response to arterial injuries [176]. Several other studies have established how a transient rise in the level of O-GlcNAcylation prevents inflammation-induced vascular dysfunction and remodeling, mainly by exerting negative feedback on NF-κB signaling [177,178,179].

Chronic O-GlcNAcylation has, instead, opposite. and deleterious effects for the vascular system. Interestingly, hypertension, vascular calcification, and vascular constriction have been associated with a chronic increase in O-GlcNAcylation levels [180,181,182]. Du et al. demonstrated that hyperglycemia induces O-GlcNAc modification of serine 1177 of eNOS. This O-GlcNAcylation occurs at the expense of phosphorylation at the same site. The result is a loss of activity of the enzyme, with consequent failure of NO production [183]. Endotheline-1 (ET-1) is an important player in the development of vascular dysfunction. O-GlcNAcylation is one mechanism through which ET-1 promotes vasoconstriction and activates a series of transcription factors leading to inflammation, oxidative stress, and tissue death [184]. Endothelial cells isolated from carotid plaques of diabetic patients and coronary arteries of diabetic mice have high levels of O-GlcNAcylation associated with reduced endothelium-dependent relaxation [128,185]. Thromboomospondine-1 (TSP-1) is a powerful pro-atherogenic protein subjected to up-regulation in DM, with a leading role in the generation of vascular wall injuries [186]. The inhibition of O-GlcNAcylation signaling inhibits TSP-1 expression in VSMC in response to high glucose [187].

Increased O-GlcNAcylation in VSMC can also contribute to vascular calcification. O-GlcNAcylation promotes osteoblastic differentiation with enhanced expression of bone-related markers such as alkaline phosphatase, osteocalcin, and bone sialoprotein via transcriptional activation of runt-related transcription factor 2 (Runx2), an osteogenic transcription factor. O-GlcNAcylation of Runx2 stimulated mineralization of extracellular matrix [188]. Furthermore, increased O-GlcNAcylation levels reduce endothelium-dependent relaxation through the overproduction of ROS via activation of NADPH oxidase [189]. A reduction in O-GlcNAcylation levels of VSMC of the aorta, on the contrary, was shown to prevent glucosamine-induced constriction [190].

#### 3.1.6. O-GlcNAcylation and Remodeling in CKD

As previously discussed, patients affected by CKD are at high risk of cardiovascular complications and cardiac remodeling. One of the several mechanisms linking CKD with cardiovascular diseases and remodeling is proteinuria, a condition often associated with both hypertension and DM [191]. Albuminuria is associated with cardiovascular morbidity and mortality in diabetics, hypertensives, and the general population, and it is known to be associated with increased left ventricular mass of the heart. Multiple pathophysiological processes, including systemic inflammation and endothelial dysfunction, may link albuminuria and proteinuria to cardiac remodeling [192]. Proteins are reabsorbed in the proximal tubule cells by receptor-mediated endocytosis, among which is megalin [193]. Megalin has a small C-terminal tail facing the cytoplasmic side and containing several domains involved in protein–protein interaction processes and serine and threonine residues highly phosphorylated by kinases. The presence of these residues testifies how the process of protein resorption in the proximal tubules is extremely dynamic and finely regulated. O-GlcNAcylation can affect this process, thus representing an additional mechanism linking proteinuria to heart diseases and remodeling. It has been widely demonstrated that in diabetic nephropathy, glomerular and tubular O-GlcNAcylation levels are dramatically increased [194]. In 2018, Pacheco and colleagues [195], by using spontaneously hypertensive rats (Shrs), demonstrated that the proteinuria observed in adult Shrs was due to the increase in O-GlcNAcylation in the renal cortex and reduction of phosphorylation. Both these mechanisms can promote the internalization of megalin from the proximal tubule luminal membranes, thus decreasing protein resorption. These data suggest that the O-GlcNAcylation can play an important role in the modulation of membrane trafficking by revealing a new regulatory mechanism of protein resorption in proximal tubule cells. Whether and how O-GlcNAcylation can affect other mechanisms linking CKD to cardiovascular remodeling needs further investigation.

## 4. Which Link between AGE-RAGE and O-Linked Glycosylation in Cardiovascular Remodeling?

OGT and OGA, the two key enzymes involved in O-linked glycosylation, are potential targets of glycation due to their protein nature. However, at present, no studies have been performed to explore how the function of these molecules is affected by AGE. The only data existing on the potential association between AGE, O-linked glycosylation, metabolic disorders, and cardiovascular remodeling emphasize the role of ROS as mediators of the detrimental effect of AGE and promoters of O-GlcNAc level increase. AGE synthesis and O-GlcNAcylation are processes occurring together in the hyperglycemic state [196]. As previously discussed, ROS are key pathogenetic factors of AGE-induced cardiac remodeling through the activation of RAGE. When cells are subjected to stress, including oxidative stress, the levels of O-GlcNAc are up-regulated [197]. The increased production of AGE and O-GlcNAcylation of nuclear factors and apoptosis are enhanced by a high-fat diet [196]. Rats fed a 12-week high-fat diet showed AGE accumulation, O-GlcNAcylation, and apoptosis, particularly in the heart. Although the study did not prove a causal relationship between AGE accumulation, O-GlcNAcylation, and the outcomes, it described the activation of different stress signaling pathways that can lead to organ damage in obesity. MG, an AGE precursor, promotes ROS generation and AGE accumulation, enhances apoptosis, and activates O-GlcNAcylation in human cardiomyocytes [31]. The increase in O-GlcNAcylation was shown to reduce ROS generation and to protect against MG-induced cell apoptosis, therefore suggesting a protective role against MG-induced damage [31,198]. The silencing of OGT by siRNA has been shown to aggravate the damaging effects induced by MG [198]. The study by Liu at al. also suggested some potential mechanisms through which O-GlcNAcylation exerts protective effects against ROS generation. These include the upregulation of antioxidant enzymes, such as superoxide dismutase and glutathione peroxidase, and the preservation of mitochondrial membrane potential [198].

Future studies will be necessary to clarify whether (1) OGA and OGT could be potential targets of glycation, (2) glycation affects OGA and OGT functions, and (3) AGEs-blockage may be effective in reducing ROS generation, O-GlcNAcylation, and cardiovascular remodeling.

## 5. Conclusions

Glycation and O-GlcNAcylation are two glucose-related pathways that can affect the function of different substrates and activate responses leading to the onset and progression of many diseases associated with altered glucose metabolism, such as cardiovascular remodeling. If, from one side, AGE synthesis is always a pathological process, the effect of O-GlcNAcylation depends on the range and duration of protein modifications, and the balance between OGT and OGA activities. Collectively, the studies discussed in the present review seem to suggest that glycation and glycosylation could be interesting therapeutic targets to prevent the damaging effects of metabolic disorders at cardiovascular levels.

Strategies to reduce AGE include AGE cross-link breakers, AGE inhibitors, RAGE antagonists, nutrition, and phytotherapy. However, only a few of them have been clinically evaluated and there are no data evaluating whether these compounds could be more effective under different conditions promoting AGE synthesis. In fact, almost all the anti-AGE therapeutic options and pharmacological substances that are under investigation have demonstrated promising results in diabetes complications in preclinical studies. However, no applications in non-diabetic conditions have been explored and the results have either not been reproduced, or only partially, in human clinical trials. Considering the pathogenic role of AGEs in the progression of cardiovascular disorders and remodeling, blockage of AGE seems to be of interest, but due to the lack of clinical confirmation, we can consider this topic simply a postulation that needs to be confirmed [199].

Regarding O-GlcNAcylation, considering that an acute increase in the levels of O-GlcNAcylation has a protective effect on acute diseases, while a chronic increase has deleterious consequences, several strategies have been hypothesized to be effective on O-GlcNAcylation tuning. Different studies showed how an acute increase in O-GlcNAcylation levels, due to pharmacological action, could play cardioprotective effects, contrasting reperfusion injuries. Several investigations carried out on different animal models have shown that the “core” of this pharmacological modulation is glutamine and/or glucosamine treatment that leads to increased O-GlcNAcylation with consequent protective action towards IR injuries [200,201]. However, other studies that used different models failed to observe the same [202]. Therefore, up to now, no convincing treatments have emerged as cardioprotective drugs by modulation of the HBP. It is well known that diabetes is associated with a chronic increase of O-GlcNAcylation levels in different cells and tissues, including cardiomyocytes, which lead to several complications, including cardiac dysfunction and myocardial I/R injury exacerbation [148,203]. Among the various studies carried out, one that deserves special mention is that of Wang et al. [154], which highlighted how, in diabetic subjects, hyperglycemia and hyperinsulinemia induced miR-24 (a key protective miRNA) reduction and aberrant O-GlcNAcylation in the diabetic heart, contributing to poor survival and increased infarct size in diabetic myocardial ischemia/reperfusion (I/R). The authors also highlight how miR-24 overexpression in murine hearts significantly reduces myocardial infarct size. miR-24 has been shown to modulate multiple key proteins including O-GlcNactransferase, ATG4A (involved in autophagy), and BIM (a pro-apoptosis protein) to protect the myocardium from I/R injury. Therefore, miR-24 could be a very suitable tool for apt regulation of O-GlcNAcylation levels and a promising therapeutic candidate for diabetic I/R injury. In conclusion, the direction in which to move regarding pharmacological strategies depends on the starting glycemic conditions and the pathologies under examination. Certainly, in non-hyperglycemic conditions and in view of a therapy aimed at protecting IR damage, treatments directed towards an increase (as controlled) of O-GlcNAcylation levels would be indicated. In contrast, therapeutic strategies aimed at reducing O-GlcNAcylation levels would certainly be more effective under hyperglycemia conditions.

## Data Availability

Not applicable.
